# Sexual Health in Adult Men with Spina Bifida

**DOI:** 10.1100/tsw.2007.191

**Published:** 2007-09-01

**Authors:** Gary W. Bong, Eric S. Rovner

**Affiliations:** Department of Urology, Medical University of South Carolina, Charleston, SC, USA

**Keywords:** spina bifida, myelomeningocele, sexuality, erectile dysfunction, fertility

## Abstract

Medical and surgical advances in the treatment of spina bifida (SB) have resulted in increasing numbers of patients reaching adulthood. As such, issues related to sexual maturity are being investigated to offer optimal healthcare to men with spina bifida. This report constitutes a review of the current literature relating to adults with spina bifida and issues of sexuality, erectile dysfunction and fertility. In general, adult males with spina bifida have normal sexual desires and an interest in addressing these issues with healthcare providers. Sexual education and access to intimacy are delayed compared to the general population. 75% of men achieve erections, but maintaining erections is a problem and some may be merely reflexive in nature. The many of these men show marked improvement with sildenafil. In SB erectile dysfunction and infertility are related to the level of neurological lesion with the best performance status in those with sacral lesions and intact reflexes. Men with lesions higher than T10 are at risk for azoospermia. There is an increased risk of neural tube defects in the children of men with spina bifida, but the current incidence with modern folic acid therapy is unknown. As the number of males with spina bifida reaching sexual maturity increases, further investigation into sexuality, sex education, intimacy, and treatments for erectile dysfunction and infertility will be needed.

## INTRODUCTION

Spina bifida (SB), a group of developmental abnormalities resulting from neural tube closure defects, affects less than 1/1000 births[1]. Medical advances over the last 4 decades, primarily surgical closure of open defects, shunting procedures and attention towards renal preservation, have markedly increased the life expectancy of these individuals. As such, there are growing numbers of men with SB reaching sexual maturity. Information regarding the sexual health of these individuals, however, is limited. Psychosexual development and sexual function are clearly affected by SB and further investigation is required to better manage males reaching puberty, starting relationships and desiring children. We review the current understanding on sexuality, erectile function and fertility in adult men with SB.

## SEXUALITY

Patients with severe congenital handicaps, including SB, often fail to develop normal sexuality due to impaired self esteem, dependence on care givers and lack of privacy[2]. Even patients with minor or inconspicuous abnormalities may have impaired or delayed sexuality secondary to issues that keep individuals from developing normal peer groups, such as urinary or fecal incontinence.

Those who are overtly handicapped, especially those who are wheelchair dependent, are often presumed to be asexual[3]. This assumption is a gross error on the part of caregivers and health care providers. Indeed, men with SB do have decreased access to sexual encounters compared to normal healthy peers, but sexual desire usually remains intact if cognitive function is normal. 80 – 100% of post-pubertal men with SB report normal sexual desires and fantasies and are actively interested in sexual activity[4,5,6]. A recent study utilizing a validated erectile function questionnaire demonstrated that 40% of men with SB engage in sexual activity[7]. These tended to be the older men (mean age 32) and those who no longer lived with their parents. Compared to the general population, initiation of sexual activity in SB males is delayed.

Sexual education is also delayed in these patients. In adult men without cognitive impairment, 23-28% are not aware of basic concepts related to intercourse and reproduction[5,8]. In the past 3 decades, the burden of sexual education has shifted from peers and parents to the classroom. This is ultimately beneficial to patients with SB who tend to have minimal peer groups, but are increasingly incorporated into the public school system. In a questionnaire-based study of 57 adult men with SB, 58% learned sexual education in school, 18% from parents, 14% from peers, 18% from books and 6% from TV, movies or other sources[8]. Although most young adults with SB are satisfied with the amount of general sex education they had received, 95% of patients and 59% of their parents stated they had inadequate knowledge of sexual health as it relates to spina bifida[9]. Only one-third had discussed sexuality issues with a doctor, but 93% and 100% of patients and parents, respectively, would discuss these issues if initiated by the health care provider.

## ERECTILE FUNCTION

The prevalence of erectile dysfunction (ED) in adult men with SB is approximately 75% based on modern criteria and is dependent upon the level of neurologic lesion[7,10]. All SB males with intact sacral reflexes and urinary continence are potentially potent. With absent sacral reflexes, the presence and severity of ED is variable. Diamond *et al.* reported that 64% of men with a lesion at T10 or lower obtained erections compared to only 14% with a lesion above T10[10]. A similar relation with neurologic level was demonstrated by Sandler who measured nocturnal penile tumescence and noted that the number of erections was related to sensory level[11]. There is some doubt with higher lesions as to whether erections are truly sexual in nature versus reflexive. Erections in individuals with lower lesions, however, appear to be initiated under normal stimuli. Hirayama demonstrated that in 22 males (mean age 24), 95% achieved erection by visual stimulation alone and 86% by tactile stimulation[6]. While the incidence of erection obtained with stimulation were high, only 27% of the patients capable of obtaining erections were satisfied with penile rigidity. Difficulty in maintaining an erection is also a major component to ED in these men[7].

As with erections, men with SB have variable ejaculation and orgasms. Up to 75% of SB men experience ejaculation[6,8,12]. This tends to be dripping in nature and may not necessarily be associated with a sexual sensation typical of orgasm. Decter *et al.* reported that 20% of men studied did not positively perceive ejaculation and this may be due, in part, to absent penile sensation[8].

## TREATMENT OF ERECTILE DYSFUNCTION

Erectile dysfunction contributes to a lack of confidence, poor body image and difficulty maintaining intimate relationships[13]. Treatment of ED in the spina bifida population is therefore important to several aspects of sexuality. These patients do not typically have anatomic abnormalities with penile architecture, as in the exstrophy-epispadias complex, and as such, often respond to conventional impotence management. Sildenafil, a Type 5 phosphodiesterase (PDE5) inhibitor, was shown to be effective in men with SB and ED[13,14]. 80% of patients responded in a dose dependent manner with the best efficacy at the 50 mg dose. Duration of erections, a primary component to ED in this population, increased 266%. One patient experienced success with the 100 mg dose after failing to see improvement in erections at the lower doses.

Neurological bypass has also been reported to result in an awareness of sensation in the penis. In one study, 3 patients received an ilioinguinal nerve transposition to the dorsal nerve of the penis with successful results by 15 months[15]. The patients noted sensation in locations where previously they reported complete anesthesia. As a result these individuals reported feeling less handicapped and as a result the penis became more integrated into their body image. Invasive surgery is not necessary for all men with SB, but the results reiterate the importance of normal penile function to male sexuality.

## FERTILITY

Infertility in SB males appears to be a common problem. Although the number of men with SB who are married and actively attempting to father a child are low, paternity rates are reported to be between 56 - 73%[8,16]. The likelihood of paternity is greater in those with lower lesions and in Decter's series, 7 of the 8 men who achieved paternity had L5 or sacral level lesions[8]. Infertility in men with higher lesions may be due to impotence and inability to ejaculate. In 10 males with impotence related to SB, electroejaculation revealed azoospermia in all patients[17]. These patients received a testicular biopsy and were found to have Sertoli cells only. The reasons for the subnormal infertility in this study are unclear. Normal testosterone production has been demonstrated in 90% of men with SB[8].

In men with SB who are fertile and father children, the risk of neural tube defects (NTD) in the offspring are greater than the general population. This risk is present whether the affected parent is the mother or the father. Older reports show a 3.7% incidence of NTD in children with SB parents. This is prior to the widespread use of prenatal folic acid, however, and may be an overestimate. Without more recent data, patients should continue to be counseled of the risk of NTDs.

## CONCLUSIONS

Many adult males with SB, regardless of severity of handicap, are interested in sexual activity and discussions related to sexuality should be initiated by heath care providers without assumptions. Erectile dysfunction is variable and dependent on the level of neurologic lesion, but the majority of these men respond to conventional treatments for ED, including PDE5 inhibitors. The prevalence of infertility in this population is unknown, especially in those with higher neurologic lesions. Further study is needed to determine if modern methods for treating azoospermia are effective in men with SB. Paternity is possible and men actively seeking to father children should be counseled about NTDs and preventative measures.
